# Molecular and Cellular Effects of CT Scans in Human Adipose Mesenchymal Stem Cells

**DOI:** 10.3390/ijms26178584

**Published:** 2025-09-03

**Authors:** Maxim Ignatov, Ekaterina E. Markelova, Anna Chigasova, Andrey Osipov, Ilia Buianov, Yuriy Fedotov, Petr Eremin, Natalia Vorobyeva, Nikolay Zyuzikov, Andreyan N. Osipov

**Affiliations:** 1 N.N. Semenov Federal Research Center for Chemical Physics, Russian Academy of Sciences, 119991 Moscow, Russia; mantroz@yandex.ru (M.I.); annagrekhova1@gmail.com (A.C.); a-2-osipov@yandex.ru (A.O.); ufedotov@mail.ru (Y.F.); nuv.rad@mail.ru (N.V.); 2 State Research Center—Burnasyan Federal Medical Biophysical Center of Federal Medical Biological Agency (SRC—FMBC), 123098 Moscow, Russia; ilyabuyanov007@gmail.com; 3 Federal Research Centre Fundamentals of Biotechnology, Russian Academy of Sciences, 119071 Moscow, Russia; markelova.ke@gmail.com; 4 MSU Institute for Artificial Intelligence, Lomonosov Moscow State University, 119192 Moscow, Russia; 5 Emanuel Institute for Biochemical Physics, Russian Academy of Sciences, 119334 Moscow, Russia; 6 National Medical Research Center for Rehabilitation and Balneology, Ministry of Health of Russia, 121099 Moscow, Russia; ereminps@gmail.com; 7 Department of Physics, Faculty of Science and Technology, The University of the West Indies, St. Augustine 999183, Trinidad and Tobago; nikolay.zyuzikov@sta.uwi.edu; 8 Veksler and Baldin Laboratory of High Energy Physics, Joint Institute for Nuclear Research, 141980 Dubna, Russia; 9 CANDLE Synchrotron Research Institute, 31 Acharyan, Yerevan 0040, Armenia

**Keywords:** DNA damage, DNA repair foci, cellular proliferation, transcriptomics, gene expression, mesenchymal stem cells, low doses, X-rays, СТ scans

## Abstract

An open question in radiobiology concerns whether low doses of radiation are harmful or if cells are able to tolerate such exposure with minimal or no disruption. This issue is relevant for evaluating public health risks associated with the increasing number of medical computed tomography (CT) diagnostic procedures. This study evaluated the impact of CT scan-level exposure on human adipose mesenchymal stem cells (hMSCs) by measuring DNA damage responses (γH2AX, 53BP1, pATM foci), proliferation (Ki-67), senescence (β-galactosidase), and multiple gene expressions. Responses to one or five CT exposures were compared to a 2 Gy X-ray dose at intervals from 1 h to 10 passages post-irradiation. It was shown that CT scan briefly increased DNA damage markers but showed no significant long-term effects. A high dose of 2 Gy X-ray exposure caused sustained DNA damage, decreased proliferation, increased senescence, and significant changes in hundreds of genes even after several cell generations. After a single CT exposure, gene expression changes were minimal, while high-dose exposure led to strong activation of DNA repair and stress response pathways. Five CT scans caused a slight activation of *LIF* and *HSPA1B* genes, but these effects were minor compared to the high-dose group. All detected effects from CT scans were not observed by ten cell passages, whereas high-dose effects persisted. In conclusion, typical CT scan exposures have only short-term, mild effects on hMSCs, while high-dose radiation causes lasting cellular and genetic changes.

## 1. Introduction

CT scans, or computed tomography, are globally common diagnostic tools [1,2,3]. Modern CT scanners produce high-resolution images and have increased their usage over traditional radiographic and other types of medical exams. Frequent high-quality CT imaging allows for faster, more accurate diagnoses and precise anatomic details for treatment planning.

CT scanners produce images by measuring X-ray attenuation from various angles [4]. The radiation dose associated with a typical CT scan is averaged from 2 to 20 mSv, depending on the examination [5]. It is comparable to the annual dose received from natural sources of radiation (1 to 10 mSv, depending on geographical location). Consequently, the health risk posed to an individual from radiation exposure during a typical CT scan is similar to that from background radiation levels. However, given the increasing number of individuals undergoing CT scans and especially the number of repeated CT scans per person, the potential public health risk may be significant. A study from 1991 to 1996 revealed that approximately 0.4% of all cancers in the United States might be attributable to radiation from CT exams based on usage data. When adjusting for levels of CT usage in 2006, it was estimated that 1.5% to 2% of cancers in the USA could eventually result from the ionizing radiation utilized in CT [6]. This was the highest estimate of cancer risk from CT scans. However, a recent study found that, given current utilization rates and radiation dose levels, CT-related cancers could potentially account for up to 5% of all new cancer diagnoses each year [7].

Children’s CT doses are much higher than adults’ (around 2 times) [8]. Moreover, in some developing countries, they could be even higher due to the use of old equipment and different protocols [9]. Cadavid (2024) also demonstrates substantial disparities and variations in pediatric CT examinations performed in multiple sites and countries [9].

Therefore, an individual single scan leads to a very low risk because of a very low dose. But the increasing number of CT scans [10], the number of procedures involving multiple scans raises concern of health risks due to high public dose [11]. Recent epidemiological studies show controversial results on the cancer risk [12,13] and may not have enough statistical power and observation time to resolve the issue. At these diagnostic-level doses, no immediate tissue injury is observed—unlike the deterministic effects seen with high-dose exposures. However, ionizing radiation can damage DNA even at low doses. X-ray photons can induce DNA damage, and while cells repair most of this damage, misrepairs can lead to cell cycle arrest, programmed cell death, loss of the cell’s ability to divide (cellular senescence), mutations, and even oncogenic transformation (cancer development) [14,15]. Among the whole spectrum of various radiation-induced DNA damages, the most critical for the further fate of the cell are double-strand breaks (DSB) of DNA [16,17]. Immunocytochemical analysis of proteins involved in the response of cells to DNA damage makes it possible to obtain unique information about post-radiation changes in the number of DNA repair sites and their distribution over the volume of the nucleus of each cell [18]. Hundreds and thousands of copies of these proteins form dynamic focal microstructures localized in the areas of DSB repair. These protein clusters, once called ionizing radiation induced foci (IRIF), are now commonly known as DNA damage foci [19,20] or DNA repair protein foci [21,22]. Among the foci-forming proteins, the most studied are H2AX phosphorylated by serine 139 (γH2AX) [23,24], 53BP1 (p53-Binding Protein 1) [25,26] and ATM (Ataxia-Telangiectasia Mutated) phosphorylated by serine 1981 (pATM) [27,28]. DNA repair errors can result in reduced cell proliferation due to cell cycle arrest or cellular senescence. Nuclei with DNA repair foci are often analysed alongside DNA repair foci using the cell proliferation marker Ki-67 (Marker of Proliferation Kiel 67) protein and cellular senescence markers, commonly aging-associated β-galactosidase (SA-β-gal) [29,30].

This paper examines how CT-equivalent exposure affects DNA repair foci, proliferation activity and the transcriptional profiles of human adipose mesenchymal stem cells (hMSCs) and their progenies (5 and 10 passages), comparing these changes to those caused by higher dose of ionizing radiation. Whole-genome RNA sequencing (RNA-seq) was conducted to analyze transcriptome profiles, allowing for the assessment of gene expression as well as the activation or suppression of diverse molecular pathways. This methodology facilitates the identification of understudied genes and cellular response pathways associated with exposure to ionizing radiation.

hMSCs are multipotent stem/stromal cells found in bone marrow, adipose and other tissues, known for their regenerative potential and contribution to tissue repair [31]. Notably, hMSCs are relatively radioresistant: they can survive and retain stem cell characteristics even after high radiation doses [31]. This resilience makes them a suitable cell type to study subtle radiation-induced changes—any transcriptional alterations are less likely to be confounded by massive cell death [32]. Moreover, hMSCs play supportive roles in tissue microenvironments (e.g., the bone marrow niche), and their radiation response could influence long-term tissue health. By analyzing DNA repair foci, cellular responses and gene expression changes 24 h after CT scan exposure and comparing them to a high dose of 2 Gy, we aimed to characterize molecular and cellular perturbations. We further investigated longer-term effects by examining cells after 10 passages following irradiation to see whether radiation exposure leaves a lasting transcriptomic “imprint” and damaging cellular effects. Understanding these CT-induced molecular and cellular changes is crucial for identifying cancer mechanisms and general low dose response. Comparing with high doses is vital for applying the Linear No-Threshold (LNT) model to estimate cancer risks based on extrapolation from high doses especially when the LNT model is incompatible with some experimental data [33].

## 2. Results

### 2.1. DNA Repair Foci

**Figure 1:**
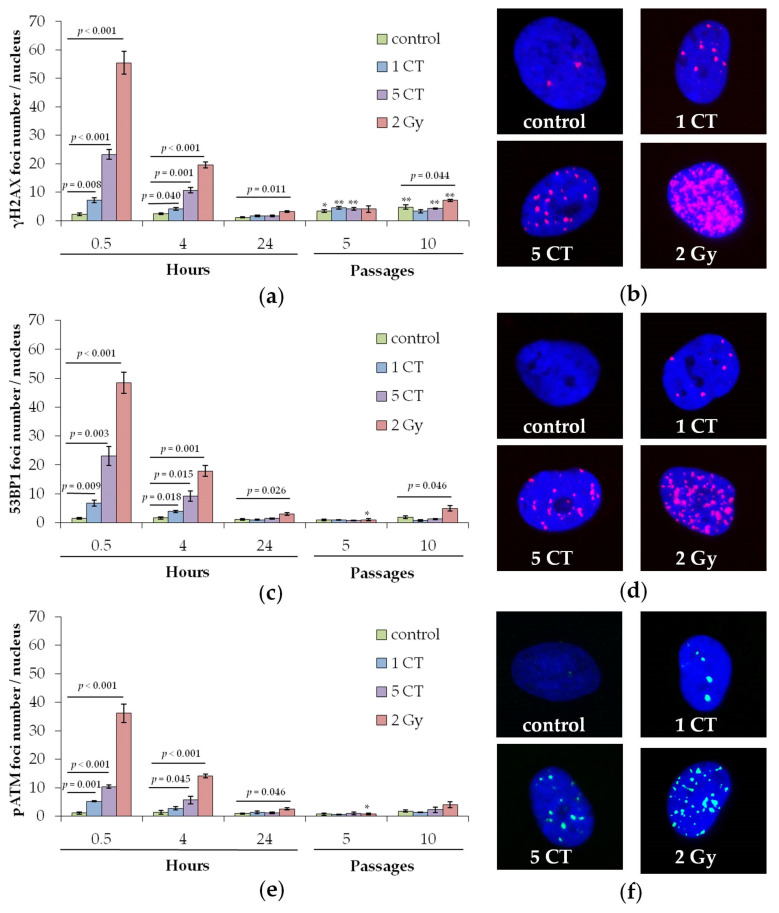
Changes in the number of DNA repair foci in hMSCs exposed to CT scans (1 and 5 CT) and X-rays (2 Gy): (**a**) γH2AX; (**c**) 53BP1; (**e**) pATM. (**b**,**d**,**f**). * *p* < 0.05, ** *p* < 0.01 compared with the corresponding 24 h samples. Representative microphotographs of immunofluorescently stained cell nuclei of hMSCs showing γH2AX ((**b**), red), 53BP1 ((**d**), green), and pATM ((**f**), green) foci, correspondingly. DAPI counterstaining is shown in blue.

Changes in the number of γH2AX foci in hMSCs at 0.5, 4, and 24 h after one CT session, five consecutive CT sessions, and X-ray irradiation at a high dose of 2 Gy, used as a positive control, are shown in Figure 1. The selection of time points was based on previous research findings, indicating that γH2AX foci in MSCs peak 0.5 h after irradiation [34], the rapid phase of DNA DSB repair concludes within 4–6 h, and the slower phase is completed by around 24 h [35].

After 2 Gy irradiation, changes in MSC foci numbers are similar to those observed for three proteins in human fibroblasts exposed to the same X-ray dose [18]. At 0.5 h post-exposure, radiation-induced foci per cell nucleus (subtracting control values) were as follows: γH2AX 53.3 ± 4.0, 53BP1 46.8 ± 3.7, and pATM 36.6 ± 3.5. Four hours after irradiation, γH2AX foci dropped to about 32%, while 53BP1 and pATM foci fell to roughly 35% of their 0.5 h levels. At 24 h post-irradiation, only 4–5% of the original foci remained. It should be noted that 24 h after irradiation with a dose of 2 Gy, the number of residual foci of γH2AX, 53BP1 and pATM proteins in MSCs was statistically significantly higher (*p* < 0.05) than the control values (Figure 1).

MSCs exposed to 88 ± 15 mGy during a single CT session exhibited a statistically significant (*p* < 0.01) increase in the number of foci for γH2AX, 53BP1, and pATM proteins observed 0.5 h post-exposure (Figure 1). The changes, compared to control values, were 5.0 ± 0.9, 5.2 ± 1.1, and 4.2 ± 0.4 foci per cell nucleus for γH2AX, 53BP1, and pATM, respectively. The levels of γH2AX and pATM are similar to the number of their respective foci observed 0.5 h after MSCs were exposed to X-rays at a dose of 80 mGy in an earlier study [34]. When MSCs undergo five sequential CT sessions with a cumulative dose of 440 mGy, the number of γH2AX, 53BP1, and pATM protein foci measured 0.5 h post-exposure increases to 21.1 ± 1.8, 21.5 ± 3.3, and 9.3 ± 0.9 foci per cell nucleus, respectively (Figure 1). Given the prolonged irradiation period, which allows some DNA repair, and an increased dose uncertainty, the protein foci counts were consistent. After 24 h of exposure, neither a single nor five consecutive CT sessions resulted in a statistically significant increase in residual γH2AX, 53BP1, or pATM foci compared to the controls (Figure 1).

The study also evaluated DNA repair protein foci in the descendants of irradiated cells at passages 5 and 10. At passage 5, DNA repair protein foci counts were comparable in irradiated and control groups (Figure 1). A statistically significant increase in the number of γH2AX and 53BP1 foci was observed in the progeny of cells irradiated with a 2 Gy dose compared to the unirradiated control group only at passage 10 (Figure 1). In contrast, only a non-significant upward trend was noted for pATM foci (*p* = 0.11). Neither exposure during a single CT session nor five consecutive CT sessions resulted in statistically significant changes in the numbers of γH2AX, 53BP1, or pATM foci in the descendants of irradiated cells at passage 10.

Studies have shown that MSCs exposed to doses equivalent to one or five CT scans had a significant rise in γH2AX, 53BP1, and pATM foci numbers 0.5 h post-irradiation. However, by 24 h and in passages 5 and 10 after irradiation, foci levels returned to control values with no significant differences.

### 2.2. Cellular Proliferation and Senescence Associated β-Galactosidase Positive Cells

Immunocytochemical analysis of Ki-67 positive cells was used to assess the proliferative activity of irradiated and control cells. The Ki-67 protein is a well-known and widely used marker of cell proliferation, as it is present in all active phases of the cell cycle (G(1), S, G(2) and mitosis) but is absent in resting cells (G(0)) [36,37].

**Figure 2:**
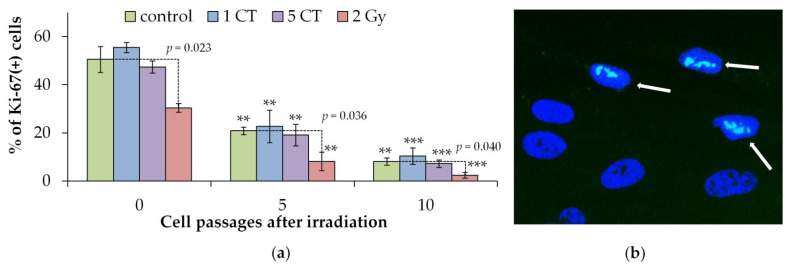
(**a**) Changes in the proportions of Ki-67(+) cells depending on the post-irradiation passage number in the control and irradiated hMSCs. ** *p* < 0.01, *** *p* < 0.001 compared with the corresponding 0 passage (24 h after exposure) samples. (**b**) Representative microphotograph of immunocytochemically labeled cells with Ki-67 antibodies (green), marked with arrows. Nuclei are counterstained with DAPI (blue).

It was shown that 24 h after irradiation (passage 0) and in passages 5 and 10, a statistically significant decrease in the proliferative activity of irradiated cells was observed only after irradiation at a dose of 2 Gy (Figure 2). One CT and five CT scan doses did not cause statistically significant changes in the proliferative activity of cells. At the same time, both control and irradiated cell populations showed a statistically significant decrease in the proliferative activity of cells with an increase in the number of cell passages.

**Figure 3:**
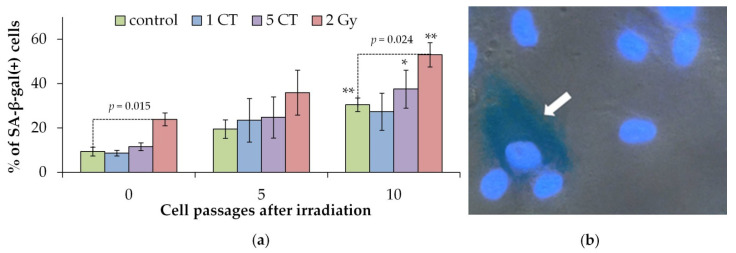
(**a**) Changes in the proportions of SA-β-gal(+) cells depending on the post-irradiation passage number in the control and irradiated hMSCs. * *p* < 0.05, ** *p* < 0.01, compared with the corresponding 0 passage (24 h after exposure) samples. (**b**) Representative image of a SA-β-gal(+) cell marked with an arrow; cytoplasm is colored dark green-blue. Nuclei are counterstained with Hoechst 33342 (light blue).

Figure 3 shows the proportion of β-galactosidase (SA-β-gal) positive cells in irradiated and control groups. Increased β-galactosidase level is a well-established marker of cellular senescence [38,39]. A significant increase in SA-β-gal positive cells was observed only after exposure to the high 2 Gy dose, compared to controls, at both 0 (24 h post-exposure) and after 10 passages (Figure 3). As cell passage number increases, there is a corresponding rise in the proportion of SA-β-gal+ cells in both control and irradiated groups. This increase generally correlates with a reduction in the proportion of proliferating Ki-67+ cells.

### 2.3. Gene Expression

**Figure 4:**
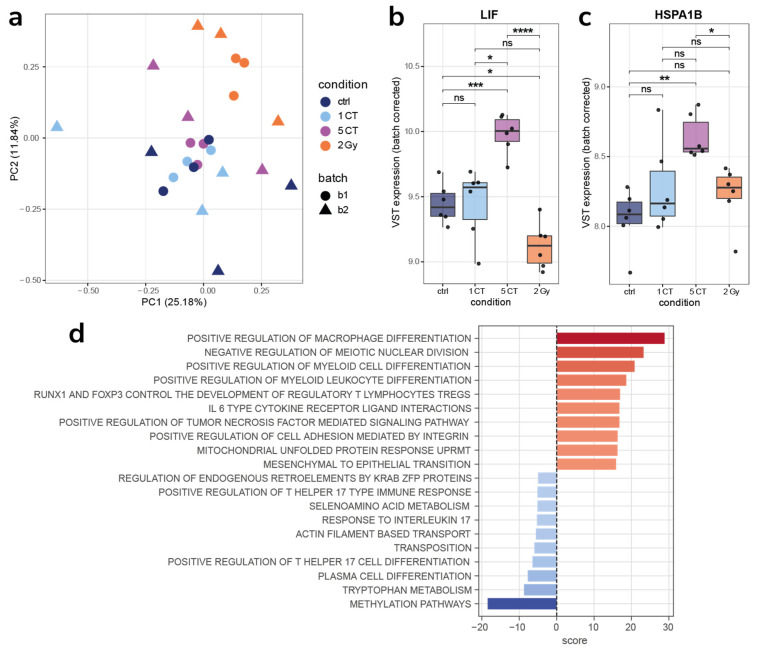
Comparison of 1 CT and 5 CT samples with 2 Gy and control samples. (**a**) PCA of batch-corrected VST-transformed counts. (**b**,**c**) Batch-corrected VST-transformed expression of *LIF* (**b**) and *HSPA1B* (**c**), which were identified as DEGs in the 5 CT vs. control comparison. (**d**) Top significantly enriched gene sets identified in 5 CT samples compared to control samples. * *p* < 0.05, ** *p* < 0.01, *** *p* < 0.001, **** *p* < 0.0001, ns, no significant.

We obtained high-quality RNA-seq data from human mesenchymal stem cells (hMSCs) exposed to one CT, five CT, and 2 Gy, as well as matched non-irradiated controls. For each condition, three biological replicates were analyzed, ensuring sufficient statistical power to detect expression changes. All samples passed quality control filters, with RNA integrity numbers (RIN) > 9 and sequencing depth averaging ~30 million paired-end reads per sample. To evaluate the overall structure of the dataset and inspect for potential batch effects or outliers, we performed principal component analysis (PCA) on the variance-stabilized transformed (VST) expression matrix (Figure 4a). PCA revealed tight clustering of biological replicates and separation of the 2 Gy samples from controls along the principal components, indicating robust transcriptomic changes at high dose. In contrast, the one CT and five CT samples clustered closely with controls, consistent with minimal perturbation to global gene expression.

**Figure 5:**
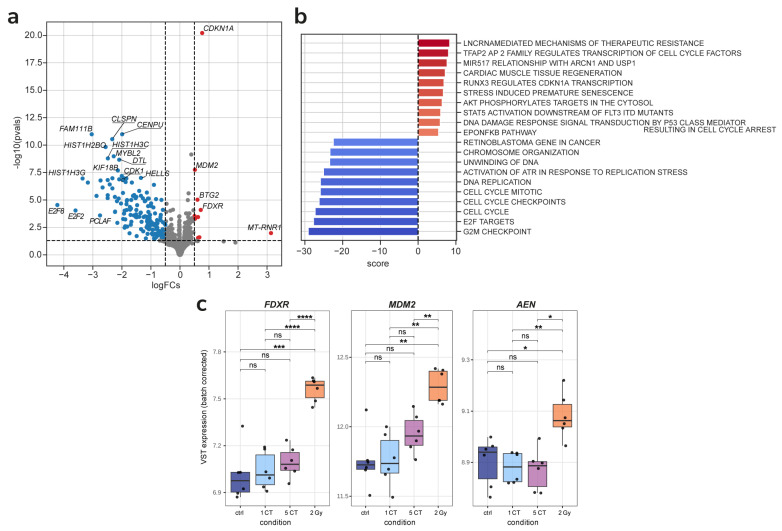
Comparison of 2 Gy samples with the control samples. (**a**) Differentially expressed genes found after 2 Gy irradiation. (**b**) Top significantly enriched gene sets identified in 2 Gy samples compared to control samples. (**c**) Batch-corrected VST-transformed expression of *FDXR*, *MDM2*, and *AEN*. * *p* < 0.05, ** *p* < 0.01, *** *p* < 0.001, **** *p* < 0.0001, ns, no significant.

After 24 h, hMSCs exposed to a single CT-equivalent dose (1 CT) showed virtually no significant changes in gene expression compared to non-irradiated controls. Using a strict FDR < 0.05 cutoff, we detected zero differentially expressed genes in the 1 CT vs. control comparison. Even when lowering the stringency (e.g., examining nominal *p* < 0.01), only a handful of genes appeared marginally up- or downregulated, and none remained significant after multiple-testing correction. Similarly, the 5 CT exposure induced only minimal transcriptomic perturbation. In the 5 CT vs. control comparison, we identified only two up-regulated genes—*LIF* (Leukemia Inhibitory Factor) and *HSPA1B* (Heat Shock Protein Family A (Hsp70) Member 1B)—at FDR ≤ 0.05 and logFC ≥ 0.5 (Figure 4b,c). This number of DEGs is dramatically small in contrast to the hundreds of genes typically altered by high-dose radiation (see Figure 5 below for 2 Gy results).

Functional analysis showed that 1 CT samples exhibited no differences from controls, aside from a small downregulation of glycogen metabolism, while 5 CT samples had more pronounced changes: activation of inflammatory pathways (Figure 4d).

Exposure to a 2 Gy dose of irradiation elicited a pronounced transcriptomic response in hMSCs, starkly contrasting the minimal changes observed after low-dose CT exposures. Differential expression analysis revealed 273 significantly altered genes (FDR < 0.05, |log_2_FC| ≥ 0.5) (Figure 5a). The activation of canonical DNA damage repair (DDR) pathways and cell cycle arrest is consistent with prior studies in irradiated cells (Figure 5b).

We also compared the expression levels of radiation-responsive genes that have even been considered as RNA markers of low-dose irradiation in previous works [40]. *FDXR* (Ferredoxin Reductase) and *MDM2* (Murine Double Minute 2) show a close gradual increase with irradiation dose, and though the difference in expression between control and 5 CT is not significant, the median expression is slightly higher. However, that is not the case for AEN (Apoptosis Enhancing Nuclease), levels of which are relatively the same in control, 1 CT, and 5 CT cells, with a high increase in 2 Gy irradiated cells.

**Figure 6:**
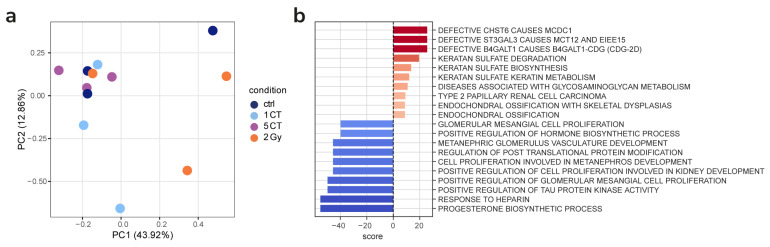
(**a**) PCA of batch-corrected VST-transformed counts from samples after 10 passages. No evident clustering of conditions. (**b**) Top significantly enriched gene sets identified in 2 Gy samples compared to control samples after 10 passages.

The same cells from the high-dose irradiation and low-dose CT scan experiments were analyzed after 10 passages. Following the removal of donor batch effects, we did not observe significant clustering of conditions in the PCA. Gene expression differences between control and high dose irradiated or CT-scanned cells were much less pronounced than after 24 h. However, we detected subtle differences in pathway activity, suggesting possible residual effects of stress in the 2 Gy-irradiated samples (Figure 6). Namely, a number of pathways associated with defective genes were upregulated.

## 3. Discussion

The validity of using standard 200 kV X-ray radiation as a positive control for CT X-rays (80–140 kVp) has been questioned. Experimental dose curves for γH2AX and 53BP1 show that, when the RBE of 200 kVp X-rays is set to 1.00, the RBE for 100 kV radiation is about 1.15—nearly within biological experiment error margins [26]. Other studies report similar RBE values (1.15–1.20) for CT X-ray radiation [41,42].

The first part of the study analyzed the radiation-induced foci involving the DNA-related proteins γH2AX, 53BP1, and pATM. Phosphorylated γH2AX (histone H2A variant, Ser139) is crucial for the cellular DNA damage response, chromatin remodeling, and recruitment of DNA repair proteins [43,44,45,46]. The tumor suppressor protein 53BP1 regulates double-strand DNA break repair and cell cycle checkpoint activation [47,48,49]. ATM is a major transducer kinase that responds to DNA damage by triggering DNA repair and activating signaling pathways [50,51,52]. Critical DNA damage, such as DSBs, primarily triggers ATM activation through autophosphorylation at serine 1981, leading to dimer dissociation [50].

Exposure to one and five CT doses significantly increased protein foci numbers only at early post-irradiation time points (0.5 and 4 h), returning to baseline by 24 h (Figure 1). No increase was detected in CT-irradiated cells at the 5th or 10th passage (Figure 1). Similar outcomes were observed in fibroblasts exposed to 100 mGy X-rays [18]. Only a high dose of 2 Gy led to a sustained rise in the foci at 24 h, and this effect persisted to passage 10, suggesting a dose threshold for DNA repair protein foci in X-ray irradiated MSCs. This persistent increase may reflect radiation-induced genomic instability and accelerated senescence, supported by reduced Ki-67+ proliferating cell proportions and increased SA-β-gal+ cells at both 24 h and passage 10 post-2 Gy. CT doses did not affect proliferation or senescence markers at any time point. Cells used in the experiments were from passages 5–6, and, by passage 10 post-irradiation, total divisions approach the Hayflick limit (each passage involves 2-3 cell divisions in average). Our data also align with studies showing that in bone marrow cells in vivo, p53 signaling is not activated by doses ≤100 mGy, while higher doses (1 Gy and 3 Gy) cause late DNA damage 30 days after irradiation [53].

Transcriptome analysis 24 h after irradiation showed that one CT scan had no statistically significant effect on gene expression, whereas five CT scans induced increased expression of *LIF* and *HSPA1B* genes. Both genes maintain cell population integrity through distinct mechanisms. The LIF protein is an interleukin class 6 cytokine that affects cell growth by inhibiting differentiation [54]. Overexpression of the *LIF* gene has been shown to increase the angiogenic potential of MSCs [55]. HSPA1B is a heat shock protein family A (Hsp70) member 1B. Proteins of the Hsp70 family are usually actively expressed in cells under stress [56]. They function as molecular chaperones inside cells and form an integrated network that is involved in a variety of processes, including folding of newly synthesized polypeptides, refolding of metastable proteins, assembly of protein complexes, dissociation of aggregates, and degradation of malformed proteins [56]. These proteins are also critical regulators of mitochondrial bioenergetics, lipid metabolism, and apoptosis, as well as innate and adaptive immune responses [56,57].

Twenty-four hours after irradiation at a dose of 2 Gy, a statistically significant change in the expression of 273 genes was observed. An increase in the expression of *MDM2*, *FDXR*, *AEN*, *CDKN1A* (Cyclin Dependent Kinase Inhibitor 1A), *BTG2* (B-Cell Translocation Gene 2), and *MT-RNR1* (Mitochondrially Encoded 12S Ribosomal RNA) genes is important. The MDM2 protein plays a crucial role in suppressing the function of the p53 protein, which acts as a “genome guardian”, promoting its ubiquitination and subsequent destruction [58,59,60]. The FDXR (Ferredoxin Reductase) flavoprotein transfers electrons from NADPH (Nicotinamide Adenine Dinucleotide Phosphate) to mitochondrial cytochrome P450 enzymes [61]. It is regulated by p53, and has recently been shown that FDXR and p53 are mutually regulated by an FDXR-p53 loop via iron homeostasis [62]. Recently, *FDXR* gene expression analysis has been used for in vivo radiation biodosimetry due to high dose-dependent upregulation in white blood cells following radiation exposure [62,63]. AEN (apoptosis-enhancing nuclease) is induced by p53 with various DNA damage, and its expression is regulated by the phosphorylation status of p53 [64]. It was shown that AEN is required for p53-dependent apoptosis [64]. It is also necessary to note the activation of the p53 signaling pathway in the DNA damage response. RUNX3 (Runt-related Transcription Factor 3) regulates CDKN1A transcription, cell cycle arrest, and stress-induced premature senescence [65]. Also noteworthy is the activation of long non-coding RNA-mediated therapeutic resistance. Long non-coding RNAs (lncRNAs) are emerging as crucial regulators of gene expression through diverse mechanisms, including regulation of protein localization, sequestration of miRNAs, recruitment of chromatin modifiers, and modulation of signaling pathways [66]. In addition to activation, suppression of various signaling pathways was also observed, in particular DNA unraveling and replication, chromosome organization, and S/G2 cell cycle control.

After 10 cell passages following exposure to both a single and five CT scans, no statistically significant differences in gene expression were detected when compared to non-irradiated controls. Notable changes relative to the control group were only observed in progeny cells irradiated with a dose of 2 Gy. Specifically, activation of signaling pathways related to keratan sulfate proteoglycan metabolism was identified. Previous studies have demonstrated that sulfation of keratan sulfate proteoglycan decreases radiation-induced apoptosis in human Burkitt’s lymphoma cell lines [67]. Among the downregulated pathways, regulation of post-translational protein modification was particularly noteworthy.

Differences were observed between CT scans and high doses of radiation across all studied outcomes, including DNA damage, senescence, proliferation, and gene expression, both in the short term and long term after irradiation. No significant differences from the control group were found following CT scans at 24 h or at later time points, indicating that any detectable effects from CT radiation do not persist beyond one day. In contrast, exposure to a high dose of 2 Gy resulted in accelerated cellular senescence and aging, as well as complex changes in gene expression detectable even several passages after exposure.

## 4. Materials and Methods

### 4.1. Cell Culture

A primary culture of MSCs from human adipose tissue of passages 5–6, obtained from the collection of Cell Systems LLC (Database: MC16.05.16, Accession numbers: 250716; 270716; 290716; 110816 and 130816, Moscow, Russia), was used. The cells were cultured in DMEM medium with 1 g/L of glucose (Thermo Fisher Scientific, Waltham, MA, USA), containing 10% fetal bovine serum (Thermo Fisher Scientific, Waltham, MA, USA) under standard CO_2_ incubator conditions (37 °C, 5% CO_2_), changing the medium every three days.

### 4.2. Irradiation

The cells were irradiated in the exponential growth phase, when the cell population density was approximately 60–70%.

A TOSHIBA AQUILION 64 CT scanner (Toshiba, Tokyo, Japan) was used to irradiate cells under parameters simulating human head scans (120 kV, 350 mA, 5 mm collimator, pitch 1). Both single and quintuple (with 5 min intervals) irradiations were conducted. Dosimetry was carried out by the thermoluminescent method using aluminum-phosphate dosimeters (by IKS-A DOSIMETRY COMPLEX (IBF, USSR, zav. No. 425)) and dosimeters based on magnesium borate (by Doza-TLD dosimetry complex (NPP Doza, Moscow, Russia)). CTDI was 86 mGy and DLP was 36,723 mGy × cm; the effective head dose was 5.1 mSv (16 cm phantom). For non-standard objects, characteristics like size, mass, and density were considered. MSCs irradiated in 35 mm petri dishes (with 2 mL culture medium volume) received absorbed doses consistent with CTDI values, confirmed by dosimetry: 88 ± 15 mGy per dish per CT session, accounting for spatial heterogeneity and detector error.

An X-ray biological facility (RUST-M1, Diagnostika-M LLC, Moscow, Russia) with two emitters was used for comparative studies and positive control. Irradiation conditions: absorbed dose 2 Gy at 0.85 Gy/min, 200 kV anode voltage, 5 mA current per tube, and a 1.5 mm Al filter. Dosimetry control of the absorbed dose was carried out by the DRK-1M clinical X-ray dosimeter (NPP Doza, Moscow, Russia). The total uncertainty of the dispensed absorbed dose did not exceed 15%.

### 4.3. Immunocytochemical Analysis

Immunocytochemical staining of cells was performed according to a previously described protocol [68]. The following primary antibodies were used: rabbit monoclonal antibodies against γH2AX (phospho S139) (dilution 1:800, clone EP854(2)Y, Abcam, Waltham, MA, USA); mouse monoclonal antibodies against 53BP1 (dilution 1:200, clone BP13, Merck-Millipore, Burlington, VT, USA); mouse monoclonal antibodies against phosphorylated ATM (phospho S1981) protein (dilution 1:400, clone 10H11.E12, Abcam, Waltham, MA, USA); mouse monoclonal antibodies against Ki-67 protein (dilution 1:400, clone Ki-S5, Merck-Millipore, Burlington, VT, USA). The following secondary antibodies were used: goat anti-mouse IgG H&L (Alexa Fluor 488 conjugated, dilution 1:2000; Abcam, Waltham, MA, USA) and goat anti-rabbit IgG H&L (Alexa Fluor^®^ 555, dilution 1:2000; Abcam, Waltham, MA, USA). Cells were imaged using a Nikon Eclipse Ni-U microscope (Nikon, Tokyo, Japan) equipped with a ProgRes MFcool high-resolution camera (Jenoptik AG, Jena, Germany), using filter sets UV-2E/C, B-2E/C, and Y-2E/C. For each data point, a total of 300–400 cells were analyzed. Foci were enumerated using DARFI software (http://github.com/varnivey/darfi; accessed on 19 September 2016) and by manual scoring.

### 4.4. Assay of β-Galactosidase-Positive Cells

To assess the proportion of β-galactosidase-positive cells, we used the commercial Cellular Senescence Assay kit (EMD Millipore, Burlington, VT, USA, Catalog Number: KAA002). The cells were stained according to the manufacturer’s protocol with minor modification, which consisted of additional staining of cell nuclei with a fluorescent DNA dye—Hoechst 33342 (Molecular Probes, Eugene, OR, USA) at a concentration of 1 μg/mL at the stage of final washing of stained cells with phosphate-buffered saline (pH 7.4). This modification made it possible to significantly improved the quality of counting β-galactosidase negative cells [69]. Visualization and documentation of microimages were performed under combined transmitted and luminescent (set of KX-U light filters: 340–380 nm excitation and 435–485 nm emission) illumination on an inverted luminescent microscope Olympus SKX 41 SF (Olympus, Tokyo, Japan), equipped with an Infinity 3-1 camera (Lumenera Corp., Ottawa, ON, Canada). A 20× objective was used. At least 200 cells per point were analyzed.

### 4.5. RNA Extraction and Sequencing

The gene expression level was evaluated by RNA-seq analyses of three biological replicates of each cell line. The InnuPREP RNA Mini Kit 2.0 together with innuPREP DNase I Digest (Analytik Jena, Berlin, Germany) were used to isolate total RNA. To measure RNA concentration, we used the Qubit 4 Fluorometer with Qubit RNA Assay kit. The RNA integrity number (RIN) was measured by TapeStation with RNA ScreenTape reagents (Agilent, Santa Clara, CA, USA). For depletion of ribosomal RNA and library construction, the KAPA RNA HyperPrep Kit with the RiboErase (HMR) kit was used. The KAPA UDI Primer Mixes were used for sample barcoding to allow their multiplexing in a single sequencing run. Library concentrations were measured using the Qubit 4 Fluorometer with the Qubit dsDNA HS Assay kit (Life Technologies, Waltham, MA, USA) and TapeStation with High Sensitivity D1000 reagents (Agilent Technologies, Inc., Santa Clara, CA, USA). The RNA sequencing was performed on the Illumina Nextseq 550 System (Illumina, Inc., San Diego, CA, USA) with reagents for single-end sequencing and a read length of 75 bp.

### 4.6. Read Mapping and Differential Expression

FASTQ read files were analyzed using the STAR software [70] in “GeneCounts” mode using transcriptome annotation from Ensembl (GRCh38 genome assembly and GRCh38.89 transcriptome annotation). In total, expression levels of 36,596 genes were measured.

We performed data normalization and differential gene expression testing using the DESeq2 R package (version 1.12.3) [71]. To account for any technical variations (e.g., slight differences between sequencing runs) (Figure S1), we included batch as a factor in the DESeq2 design model for differential expression. This inherently performs a batch effect correction by adjusting the estimates of expression differences for batch-to-batch variability. Differentially expressed genes (DEGs) between irradiated samples and control (0 Gy) were identified for each condition (1 CT vs. control, 5 CT vs. control, 2 Gy vs. control). DESeq2 uses shrinkage estimators for dispersion and fold change; genes with an adjusted *p*-value (false discovery rate, FDR) < 0.05 were considered significantly differentially expressed. We also applied a modest fold-change threshold (|log_2_FC| ≥ 1) to focus on biologically meaningful changes. Quality control plots (MA-plots, dispersion estimates) were examined to ensure model assumptions were met. Principal component analysis (PCA) was performed on the variance-stabilized expression data to visualize global transcriptomic differences between conditions.

### 4.7. Pathway and Transcription Factor Analysis

To interpret the functional significance of the expression changes, we carried out enrichment and regulator analyses using the decoupler framework. The decoupler package provides an ensemble of computational methods to infer biological activities from omics data, leveraging prior knowledge databases. We utilized decoupler to identify (a) enriched pathways/gene sets and (b) key upstream transcription factor (TF) activities associated with the observed gene expression changes. For pathway analysis, we tested gene sets from curated databases (such as Reactome and Gene Ontology) for enrichment in the up- or downregulated genes of each condition. For transcription factor analysis, we used decoupler’s implementation of the Dorothea regulon collection (via the OmniPath database) to estimate the activity of transcriptional regulators from the expression of their target genes. In practice, decoupler was applied to the matrix of log2 fold changes, using the analytic rank-based enrichment (auRSS) method for pathways and the viper algorithm for TF activity inference—these methods combine the expression changes of genes in a regulon to infer whether the regulator is likely activated or inhibited. The significance of enrichment or activity scores was assessed by permutation-based *p*-values provided by decoupler. Results were filtered at an FDR < 0.1 for pathway and TF analyses, given the lower power in these tests. All analyses were conducted in R (v4.1) and Python (v3.9) environments.

### 4.8. Statistical Analysis

Statistical data analyses were performed using Statistica 8.0 software (StatSoft, Tulsa, OK, USA). Results are presented as the means of three independent experiments ± standard error (SE).

## Data Availability

The data used to support the findings of this study are available from the corresponding author upon request.

## Supplementary Materials

The following supporting information can be downloaded at: https://www.mdpi.com/article/10.3390/ijms26178584/s1.
