# Supplementary figures and images for: Molecular and Cellular Effects of CT Scans in Human Adipose Mesenchymal Stem Cells
Source: Int J Mol Sci. 2025 Sep 3;26(17):8584. doi: 10.3390/ijms26178584 (PMC12428932; doi:10.3390/ijms26178584)

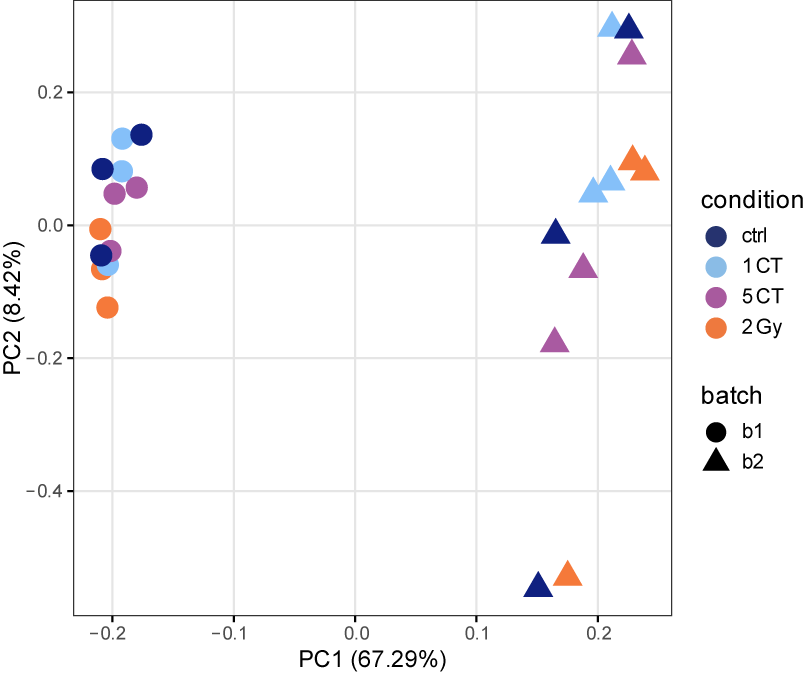

Supplement: Supplementary file 1 [file ijms-26-08584-s001.zip › Figure S1.tif]
